# Association between soy product consumption, duration of physical exercise, and psychological symptoms among Tibetan college students: a cross-sectional study in high-altitude regions of China

**DOI:** 10.3389/fpsyg.2025.1697360

**Published:** 2025-11-12

**Authors:** Yufeng Zhang, Baofeng Liu, Zhi Li

**Affiliations:** 1 School of Physical Education, Nanyang Normal University, Nanyang, China; 2 School of Physical Education and Health, Nanyang Vocational College of Agriculture, Nanyang, China; 3 School of Information Engineering, Nanyang Institute of Technology, Nanyang, China

**Keywords:** soy product consumption, duration of physical exercise, psychological symptoms, Tibetan, high-altitude regions

## Abstract

**Background:**

The prevalence of psychological symptoms among college students continues to rise, becoming a significant public health issue worldwide. The occurrence of psychological symptoms is closely associated with dietary behaviors and physical exercise. However, few studies have examined the association between soy product consumption, duration of physical exercise, and psychological symptoms among Tibetan college students in high-altitude regions.

**Methods:**

This study employed stratified cluster sampling to conduct a cross-sectional questionnaire survey on soy product consumption, duration of physical exercise, and psychological symptoms among 7,070 Tibetan college students aged 19–22 in China’s high-altitude regions. Associations among these variables were analyzed using univariate analysis, binary logistic regression analysis, and generalized linear model-based binary logistic regression analysis.

**Results:**

Among Tibetan college students in China’s high-altitude regions, the proportions consuming soy products consumption ≤2 times/day, 3–5 times/day, and ≥5 times/day were 38.7, 40.2, and 21.1%, respectively. Duration of physical exercise was <30 min/day, 30–60 min/day, and >60 min/day in 74.2, 17.9, and 8.0% of participants, respectively. The prevalence of psychological symptoms among Tibetan college students in China’s high-altitude regions was 16.6%. The prevalence of psychological symptoms was lower among boys (14.6%) than girls (18.2%), with a statistically significant difference (*χ*^2^ = 16.622, *p <* 0.001). Adjusted binary logistic regression analysis using generalized linear models showed that, with the group consuming soy products consumption ≥5 times/day and duration of physical exercise >60 min/day as the reference group, the group with soy product consumption ≤2 times/day and duration of physical exercise <30 min/day had the highest risk of depressive symptoms (OR = 4.32, 95% CI: 2.49–7.51) (*p <* 0.001).

**Conclusion:**

There is an association between soy product consumption, duration of physical exercise, and psychological symptoms among Tibetan college students in China’s high-altitude regions. Those with a higher frequency of soy product consumption and longer duration of physical exercise exhibit lower prevalence of psychological symptoms. Future prevention and intervention strategies for psychological symptoms should incorporate soy product consumption and duration of physical exercise as factors to better promote the mental health development of Tibetan college students.

## 1 Introduction

With the rapid shift in lifestyles, prolonged periods of light physical activity and screen time, excessive consumption of sugary beverages, reduced physical exercise, and extended sedentary behavior have led to a persistent rise in the prevalence of psychological symptoms among college students (Almoraie et al., 2025). Compounded by the dual pressures of employment and academic demands, college students experience declining sleep quality, irregular daily routines, and disordered eating habits—all significant factors contributing to the persistent rise in the prevalence of psychological symptoms (Jurado-Gonzalez et al., 2025). A cross-national study revealed that among college students across multiple European countries, the overall prevalence of high stress, depression, and generalized anxiety symptoms ranged from 30 to 61.30% (O'Neill et al., 2018). Research also indicates that among American college students, the prevalence rates of depression, despair, and suicidal thoughts stand at 18.4, 12.5, and 6.4% respectively, with psychological symptoms showing an upward trend (Garlow et al., 2008). Similarly, developing countries are no exception. A survey of Chinese college students revealed that the prevalence of psychological symptoms among them ranges from 14.6 to 48.6%, showing a persistent upward trend (Ochnik et al., 2021). Research also indicates that the prevalence of psychological symptoms among Chinese college students reaches as high as 17.31%, exerting a certain negative impact on academic performance and future achievements (Zhao et al., 2023). Research has also found that psychological symptoms emerging during college years may persist into adulthood, increasing the risk of developing various mental health disorders in adulthood (Alkhawaldeh et al., 2023). Therefore, effectively preventing the occurrence of psychological symptoms among college students holds significant practical importance. Multiple studies have confirmed that the emergence of psychological symptoms in college students is influenced by a combination of factors, with dietary behaviors and exercise habits having the most pronounced impact (Deasy et al., 2014; Xiang et al., 2025). Effectively analyzing the factors influencing the occurrence of psychological symptoms among college students is particularly crucial for preventing their emergence.

Soy products, rich in various nutrients essential for the body, offer numerous benefits for the physical and mental health of college students (Huang et al., 2024). Research indicates that soy products are rich in high-quality proteins, whose amino acid composition most closely matches human requirements. Additionally, they contain no cholesterol, making them highly beneficial for college students’ health (Qiu et al., 2024). A study of Chinese adults found that compared to participants who consumed soy foods less than once per week, those who consumed soy foods ≥4 times per week had a multiple-corrected odds ratio of 0.638 for low grip strength. This indicates that habitual consumption of higher amounts of soy foods is positively associated with grip strength scores reflecting muscle strength, suggesting that soy food consumption may benefit muscle health (Wu et al., 2023). Additionally, research indicates that soy products are rich in dietary fiber, which effectively promotes colonic motility, prevents constipation, and exerts positive effects on intestinal health (Ren et al., 2024). Research has also found that soy product consumption can effectively prevent the onset of cardiovascular and cerebrovascular diseases as well as chronic diseases, playing a positive role in the prevention of cardiovascular and cerebrovascular diseases (Deng et al., 2024). Meanwhile, the soy isoflavones in soy products exert a positive influence on regulating bodily hormones and providing antioxidant properties, thereby playing a beneficial role in mood regulation (Yin et al., 2024). Another study of Japanese adults showed that for each standard deviation increase in total legume intake, total soy intake, and total soy isoflavone intake, the multivariable-adjusted odds ratios for cognitive impairment were 0.48, 0.51, and 0.55, respectively. This suggests that overall soy and soy isoflavone intake may reduce the risk of cognitive impairment in adults (Nakamoto et al., 2018). However, the positive relationship between soy product consumption and health is not entirely consistent. A study involving 13,760 adults found a J-shaped relationship between the frequency of soy food intake and the incidence of depressive symptoms. This research provides the first evidence suggesting that mild to moderate consumption of soy foods may reduce the incidence of depressive symptoms, while relatively high intake may produce the opposite effect (Yu et al., 2018). This indicates that previous studies have yielded inconsistent findings. Furthermore, past research has primarily focused on the relationship between soy product consumption and cancer, cardiovascular and cerebrovascular diseases, and gastrointestinal disorders, while studies examining the association between soy product consumption and psychological symptoms among college students remain scarce (Lee et al., 2014). Additionally, no studies have yet been identified examining the association between soy product consumption and psychological symptoms among Tibetan college students in high-altitude regions.

In addition to the link between legume consumption and health, research on the duration of physical exercise and health is also increasing. Studies show that maintaining 30 to 60 min of daily physical activity has a positive effect on participants’ mental health (Wilhite et al., 2023). Another study of Norwegian college students revealed a dose–response relationship between physical inactivity and poor mental health, self-harm, and suicide attempts. The duration and intensity of physical exercise were significantly associated with mental health issues, though typically linked to smaller odds ratios (Grasdalsmoen et al., 2020). Research has also found that an appropriate duration of physical exercise can promote the maintenance of a stable and balanced gut microbiota environment, exerting a positive effect on brain hormone secretion. The gut-brain axis theory further indirectly explains the relationship between active exercise and mental health (Camara-Calmaestra et al., 2022). Another study also found that engaging in more than 60 min of moderate-to-vigorous physical activity daily can stimulate the release of dopamine and serotonin in the body and brain, exerting a positive impact on preventing psychological symptoms and maintaining overall health (Bjorkman and Ekblom, 2022). However, conclusions regarding the mental health benefits of physical exercise are not entirely consistent. A study of college students found that the positive effects of exercise on mental health showed a declining trend when the duration of physical exercise exceeded 60 min (Ribeiro et al., 2023). This indicates the necessity of researching the relationship between the duration of physical exercise and psychological symptoms among college students to further validate the association between these two factors. Notably, past studies have primarily focused on the relationship between physical exercise and mental health among college students in China’s eastern plains, with few studies conducted on Tibetan college students in high-altitude regions.

Previous studies have consistently demonstrated a strong association between soy product consumption, duration of physical exercise, and psychological symptoms. Notably, no research has yet examined the combined effect of soy product consumption and duration of physical exercise on psychological symptoms. The occurrence of psychological symptoms among college students is influenced by multiple interrelated factors, making it necessary to analyze this combined effect. Additionally, previous studies targeting the general adult population have yielded inconsistent findings, making this area worthy of further exploration and research. However, previous research has mainly focused on college students in plains regions or the developed eastern parts of China, with little attention given to Tibetan college students in the less-developed, high-altitude western regions (Yu et al., 2023). The Qinghai-Tibet Plateau region of China is a classic high-altitude area worldwide. Due to its harsh natural environment and thin oxygen levels, this region has certain negative impacts on the physical and mental health development of Tibetan college students in the area (Zhao et al., 2024). In addition, it may also be affected by the special environment in high-altitude areas, resulting in certain differences in the factors affecting the psychological symptoms of Tibetan college students and those in plain areas. To this end, this study focuses on Tibetan college students in the region. Through a cross-sectional survey, it analyzes the associations among soy product consumption, duration of physical exercise, and psychological symptoms. The research aims to provide insights for the prevention and intervention of psychological symptoms among Tibetan college students in high-altitude areas.

## 2 Methods

### 2.1 Participants

**Figure 1:**
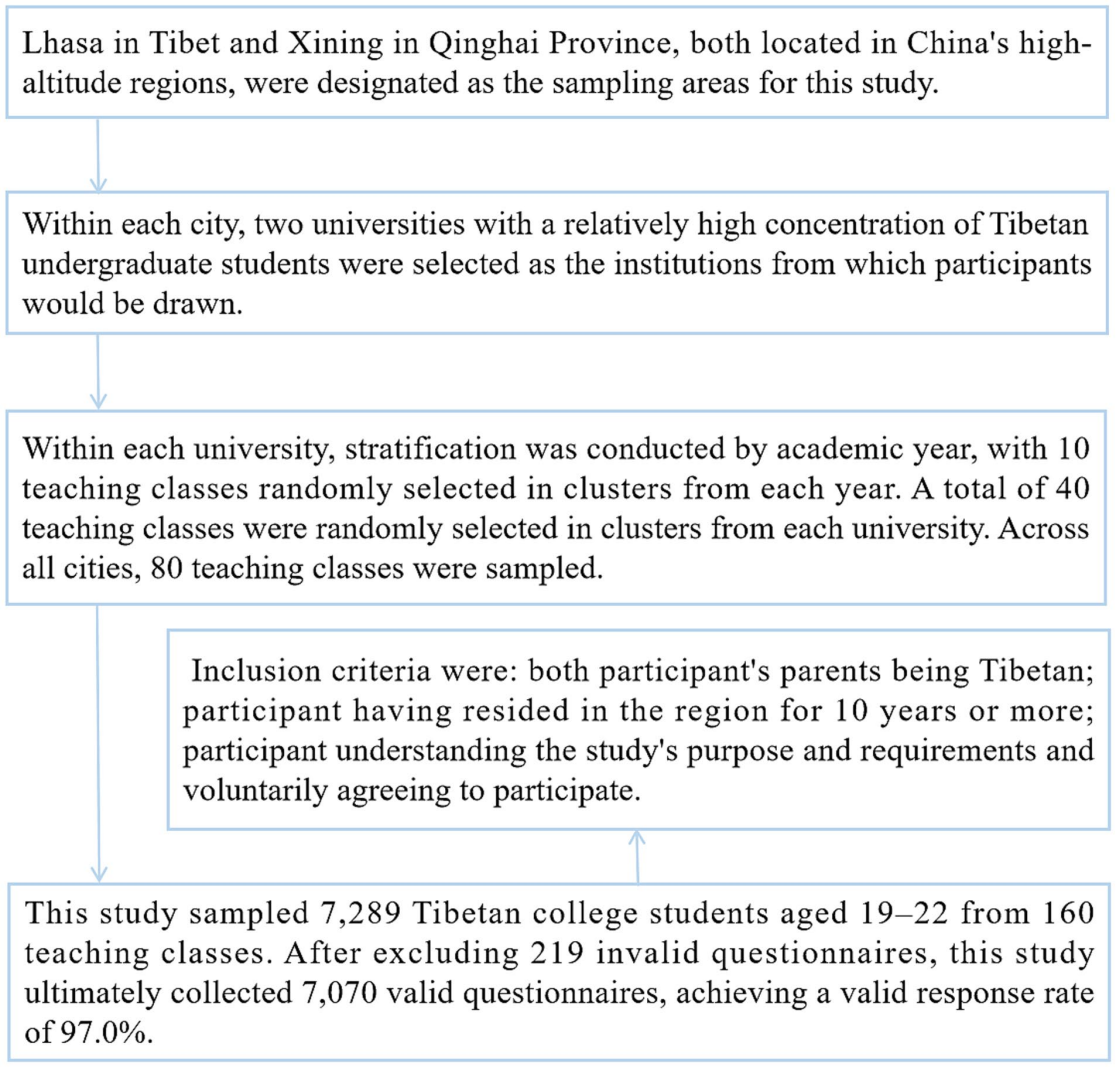
Sampling procedure for Tibetan college students in China’s high-altitude regions.

Participants in this study were selected using stratified cluster sampling, following these specific steps. This research adopted a multi-stage sampling approach conducted across high-altitude regions of China. Lhasa in Tibet and Xining in Qinghai Province were chosen as the primary sampling locations. Within each city, two universities with substantial populations of Tibetan undergraduate students were selected. The sampling process further involved stratification by academic year, with 10 classes randomly cluster-sampled from each year per university. This resulted in the selection of 40 classes per institution, totaling 80 classes across both cities. The final sample comprised 7,289 Tibetan college students aged 19 to 22, drawn from 160 academic classes. All students within each class meeting the inclusion criteria participated in the cross-sectional questionnaire survey. Inclusion criteria were: both participants’ parents being Tibetan; participant having resided in the region for 10 years or more; participant understanding the study’s purpose and requirements, and voluntarily agreeing to participate. After excluding 219 invalid questionnaires, this study ultimately collected 7,070 valid questionnaires, achieving a valid response rate of 97.0% (Figure 1).

This study was conducted by the principles of the Declaration of Helsinki. Written assent was obtained from parents/guardians. The participants also signed up for the study on their own. Approved by the Human Ethics Committee of Nanyang Normal University (8497231).

### 2.2 Assessment of psychological symptoms

This study employed the Multidimensional Sub-health Questionnaire for Adolescents (MSQA) to assess psychological symptoms among Tibetan college students in China’s high-altitude regions (Xu et al., 2020). The MSQA questionnaire is effective for assessing psychological symptoms among Chinese college students. It demonstrates good reliability and validity, with a Cronbach’s *α* coefficient of 0.957, test–retest reliability of 0.868, and split-half reliability of 0.942. The criterion-related validity of the MSQA with the SCL-90 and CMI questionnaires was 0.636 and 0.649, respectively, indicating its effectiveness in assessing sub-health conditions among adolescents (Yang et al., 2022; Zhang et al., 2025). At the same time, this scale has also been investigated among college students in high-altitude areas and has good reliability and validity (Song et al., 2024). This questionnaire consists of 15 items organized into three dimensions: emotional symptoms, behavioral symptoms, and social adaptation difficulties. Each item primarily assesses participants’ specific experiences over the past 3 months. Examples include “Frequently feeling troubled by minor matters” and “Constantly feeling that others are working against me.” Each item is categorized based on the duration of the behavior: “lasting more than 3 months,” “lasting more than 2 months,” “lasting more than 1 month,” “lasting more than 2 weeks,” “lasting more than 1 week,” or “not present or lasting less than 1 week,” corresponding to levels 1–6, respectively. Participants scoring levels 1–3 receive 1 point, while levels 4–6 receive 0 points. The total questionnaire score ranges from 0 to 15 points. Participants scoring ≥7 points are assessed as exhibiting psychological symptoms. The cutoff criteria for the three dimensions—emotional symptoms, behavioral symptoms, and social adaptation difficulties—are ≥4 points, ≥1 point, and ≥2 points, respectively.

### 2.3 Assessment of soy product consumption

This study employed a self-report questionnaire to assess soy product consumption among Tibetan college students in China’s high-altitude regions. The questionnaire primarily evaluated participants’ consumption of soy products over the preceding seven days. The questionnaire development process was guided by relevant literature (Duan et al., 2022). The evaluation of soy products in this study primarily includes tofu, bean paste, dried tofu, soy-based snacks, soy foods, soy milk, and soy beverages. Based on the study’s methodology, participants’ soy product consumption was categorized into three groups: ≤2 times/day, 3–5 times/day, and ≥5 times/day.

### 2.4 Assessment of duration of physical exercise

This study employed the China National Survey on Students’ Constitution and Health (CNSSCH) questionnaire to assess the duration of physical exercise among Tibetan college students in China’s high-altitude regions (CNSSCH Association, 2022). The CNSSCH is China’s largest nationwide adolescent health assessment initiative, conducted every 5 years across the entire country. It evaluates 30 indicators, including physiological indicators, body composition indicators, physical function indicators, and physical fitness indicators. The assessment of “duration of physical exercise” in this study primarily refers to the cumulative time participants engaged in MVPA over the preceding 7 days. MVPA encompasses multiple activities, including ball sports, walking, lifting heavy objects, housework, running, cycling, ice skating, skiing, and net sports. MVPA is defined as activities that induce sweating. This study assessed participants’ specific activity duration over the past 7 days, encompassing weekdays (Monday to Friday) and weekends (Saturday and Sunday). By surveying exercise duration and frequency, the average MVPA duration over the past 7 days was calculated. Drawing on previous studies, MVPA was categorized into three groups: <30 min/day, 30–60 min/day, and >60 min/day (Ajja et al., 2021; Zhang et al., 2020).

### 2.5 Covariates

The covariates in this study include BMI, socioeconomic status (SES), breakfast frequency, sleep quality, and screen time. BMI was calculated based on participants’ height and weight using the formula: weight (kg) / height (m)^2^. Height and weight measurements were assessed according to the requirements and standards of the Chinese National Center for Health Statistics (CNSSCH). Height was measured to the nearest 0.1 centimeter, and weight to the nearest 0.1 kilogram. BMI classifications follow the Chinese adult BMI classification standards. In this study, BMI < 18.5 kg/m^2^ is categorized as “slimmer,” 18.5–23.9 kg/m^2^ as “normal,” 24.0–27.9 kg/m^2^ as “overweight,” and ≥ 28.0 kg/m^2^ as “obese”(Wang et al., 2021). Socioeconomic status (SES) was primarily assessed through participant self-report questionnaires. Participants’ family socioeconomic status was evaluated based on parental occupation, parental educational attainment, household income, and household appliance ownership. Occupational classification followed the scoring criteria of the Ganzeboom International Socioeconomic Status Occupational Classification Index (ISEI) (Ganzeboom and Treiman, 1996). This study calculated SES using the methodology referenced from the 2003 Programme for International Student Assessment (PISA) (Turner and Adams, 2007). Higher SES scores indicate higher socioeconomic status among participants’ households. Total scores ranged from −4.98 to 4.03. Participants were categorized into percentiles based on SES scores: Low (<P25), Medium (P25-75), and High (>P75). Breakfast frequency was primarily based on participants’ breakfast consumption over the past week. This study categorized breakfast intake as ≤2 times/week, 3–5 times/week, or ≥6 times/week. Sleep quality was assessed using the Pittsburgh Sleep Quality Index (PSQI). This 7-item scale scores each dimension from 0 to 3, yielding a total score ranging from 0 to 21, where higher scores indicate poorer sleep quality. Participants scoring ≤5 were classified as having “Good” sleep, while scores >5 indicated “Poor” sleep quality. This scale is widely used among Chinese adolescents. Screen time primarily assessed participants’ average daily duration of mobile phone, tablet, laptop, and television use over the past 7 days. Based on average daily screen time and classification criteria from previous studies, participants were categorized as ≤2 h/day or >2 h/day.

### 2.6 Statistical analysis

This study conducted a cross-sectional self-report questionnaire survey on soy product consumption, duration of physical exercise, and psychological symptoms among 7,070 Tibetan college students aged 19–22 in China’s high-altitude regions. The proportions of different categories of Tibetan college students were presented as percentages. The comparison of psychological symptom detection rates among Tibetan college students with varying soy product consumption and physical exercise duration was performed using chi-square tests. The association between soy product consumption, duration of physical exercise, and psychological symptoms among Tibetan college students was analyzed using binary logistic stratified regression. After stratification by sex, binary logistic regression analyses were conducted with the presence of psychological symptoms as the dependent variable and different levels of soy product consumption and duration of physical exercise as independent variables. Model 1 did not adjust for any covariates. Model 2 adjusted for age, SES, and BMI. Model 3 further adjusted for breakfast frequency, sleep quality, and screen time based on Model 2. To further analyze the association between the combined effects of soy product consumption and duration of physical exercise and psychological symptoms among Tibetan college students in high-altitude areas, this study employed a binary logistic regression analysis using generalized linear models. After stratifying by sex, a binary logistic regression analysis using a generalized linear model was conducted. The dependent variable was the presence of psychological symptoms among Tibetan college students, while the independent variables comprised different combinations of soy product consumption and duration of physical exercise. The model adjusted for age, SES, BMI, breakfast frequency, sleep quality, and screen time. The logistic regression results reported odds ratios (OR) and 95% confidence intervals (CI).

Data analysis in this study was performed using SPSS 25.0 software (SPSS Inc., Chicago, IL, USA). Images were generated using GraphPad Prism 8.0 software. A two-tailed significance level of *α* = 0.05 was adopted.

## 3 Results

This study the average age of participants was (20.17 ± 1.03) years. Participants’ height, weight, and BMI were (168.53 ± 8.33) cm, (68.35 ± 22.31) kg, and (23.31 ± 6.24) kg/m^2^, Differences between sexes were statistically significant (*t* = 95.690, 25.587, 6.973, *p <* 0.001). The results of this study indicate that among Tibetan college students in high-altitude regions of China, the proportions consuming soy products ≤2 times/day, 3–5 times/day, and ≥5 times/day were 38.7, 40.2, and 21.1%, respectively, comparisons between sex showed statistically significant differences (χ^2^ = 19.941, *p <* 0.001). The proportions of physical exercise duration being <30 min/day, 30–60 min/day, and >60 min/day were 74.2, 17.9, and 8.0%, respectively. Comparing these proportions between sexes revealed a statistically significant difference (*χ*^2^ value = 197.810, *p <* 0.001). The prevalence of psychological symptoms among Tibetan college students in China’s high-altitude regions was 16.6%. Regarding different dimensions, the proportions of emotional symptoms, behavioral symptoms, and social adaptation difficulties among Tibetan college students in China’s high-altitude regions were 17.9, 18.0, and 14.7%, respectively. Comparison results for other categories are shown in Table 1.

**Table 1:** Basic profile of Tibetan college students in China’s high-altitude regions.

Items	Boys	Girls	*χ*^2^/t-value	*p*-value	Total
Number	3,119	3,951			7,070
Age (years)	20.30 ± 1.07	20.07 ± 0.98	9.384	<0.001	20.17 ± 1.03
Height	175.57 ± 6.06	162.97 ± 5.01	95.690	<0.001	168.53 ± 8.33
Weight	75.66 ± 21.57	62.58 ± 21.16	25.587	<0.001	68.35 ± 22.31
BMI	23.89 ± 5.76	22.85 ± 6.56	6.973	<0.001	23.31 ± 6.24
BMI classification			289.124	<0.001	
Slimmer	334 (10.7)	831 (21.0)			1,165 (16.5)
Normal	1,615 (51.8)	2,132 (54.0)			3,747 (53.0)
Overweight	592 (19.0)	306 (7.7)			898 (12.7)
Obese	578 (18.5)	682 (17.3)			1,260 (17.8)
Socioeconomic status (SES)			14.006	0.001	
Low (<*P*_25_)	533 (17.1)	548 (13.9)			1,081 (15.3)
Medium (*P*_25-75_)	2,158 (69.2)	2,848 (72.1)			5,006 (70.8)
High (>*P*_75_)	428 (13.7)	555 (14.0)			983 (13.9)
Breakfast frequency			399.885	<0.001	
≤2 times/week	600 (19.2)	325 (8.2)			925 (13.1)
3–5 times/week	1,084 (34.8)	910 (23.0)			1994 (28.2)
≥6 times/week	1,435 (46.0)	2,716 (68.7)			4,151 (58.7)
Sleep quality			20.053	<0.001	
Good (≤5 points)	937 (30.0)	998 (25.3)			1935 (27.4)
Poor(>5 points)	2,182 (70.0)	2,953 (74.7)			5,135 (72.6)
Screen Time			14.259	<0.001	
≤2 h/day	880 (28.2)	958 (24.2)			1838 (26.0)
>2 h/day	2,239 (71.8)	2,993 (75.8)			5,232 (74.0)
Soy product consumption			19.941	<0.001	
≤2 times/day	1,130 (36.2)	1,606 (40.6)			2,736 (38.7)
3–5 times/day	1,268 (40.7)	1,575 (39.9)			2,843 (40.2)
≥5 times/day	721 (23.1)	770 (19.5)			1,491 (21.1)
Duration of physical exercise			197.810	<0.001	
<30 min/days	2062 (66.1)	3,181 (80.5)			5,243 (74.2)
30–60 min/day	761 (24.4)	503 (12.7)			1,264 (17.9)
>60 min/days	296 (9.5)	267 (6.8)			563 (8.0)
Psychological symptoms					
Emotional symptoms	490 (15.7)	776 (19.6)	18.317	<0.001	1,266 (17.9)
Behavioral symptoms	507 (16.3)	768 (19.4)	11.946	0.001	1,275 (18.0)
Social adaptation difficulties	449 (14.4)	590 (14.9)	0.401	0.526	1,039 (14.7)
Psychological symptoms	455 (14.6)	720 (18.2)	16.622	<0.001	1,175 (16.6)

BMI, Body mass index.

Table 2 presents a comparison of the prevalence rates of psychological symptoms among Tibetan college students in different categories across China’s high-altitude regions. Results indicate that among Tibetan college students in China’s high-altitude regions, the prevalence rates of psychological symptoms were 20.8, 16.0, and 10.1% for groups consuming soy products ≤2 times/day, 3–5 times/day, and ≥5 times/day, respectively. These differences were statistically significant (*χ*^2^ = 81.274, *p <* 0.001). Among Tibetan college students in China’s high-altitude regions, the prevalence rates of psychological symptoms were 19.4, 8.8, and 8.7% for groups with physical exercise durations of <30 min/day, 30–60 min/day, and >60 min/day, respectively. These differences were statistically significant (*χ*^2^ = 109.892, *p <* 0.001). Comparison results for psychological symptom prevalence among other categories of Tibetan college students are shown in Table 2.

**Table 2:** Comparative study on the prevalence of psychological symptoms among different categories of Tibetan college students in China’s high-altitude regions.

Items	No psychological symptoms	Psychological symptoms	*χ*^2^/t-value	*p*-value
Number	5,895 (83.4)	1,175 (16.6)		
Sex			16.622	<0.001
Boys	2,664 (85.4)	455 (14.6)		
Girls	3,231 (81.8)	720 (18.2)		
BMI classification			14.144	0.003
Slimmer	948 (81.4)	217 (18.6)		
Normal	3,150 (84.1)	597 (15.9)		
Overweight	774 (86.2)	124 (13.8)		
Obese	1,023 (81.2)	237 (18.8)		
Socioeconomic status (SES)			64.341	<0.001
Low (<*P*_25_)	811 (75.0)	270 (25.0)		
Medium (*P*_25-75_)	4,248 (84.9)	758 (15.1)		
High (>*P*_75_)	836 (85.0)	147 (15.0)		
Breakfast frequency			178.654	<0.001
≤2 times/week	702 (75.9)	223 (24.1)		
3–5 times/week	1,526 (76.5)	468 (23.5)		
≥6 times/week	3,667 (88.3)	484 (11.7)		
Sleep quality			116.438	<0.001
Good (≤5 points)	1764 (91.2)	171 (8.8)		
Poor(>5 points)	4,131 (80.4)	1,004 (19.6)		
Screen Time			60.138	<0.001
≤2 h/days	1,639 (89.2)	199 (10.8)		
>2 h/days	4,256 (81.3)	976 (18.7)		
Soy product consumption			81.274	<0.001
≤2 times/day	2,166 (79.2)	570 (20.8)		
3–5 times/day	2,389 (84.0)	454 (16.0)		
≥5 times/day	1,340 (89.9)	151 (10.1)		
Duration of physical exercise			109.892	<0.001
<30 min/days	4,228 (80.6)	1,015 (19.4)		
30–60 min/day	1,153 (91.2)	111 (8.8)		
>60 min/days	514 (91.3)	49 (8.7)		

BMI, Body mass index.

Table 3 presents the results of univariate analyses examining the relationship between soy product consumption, duration of physical exercise, and psychological symptoms among Tibetan college students in China’s high-altitude regions. Results indicate statistically significant differences in the prevalence rates of emotional symptoms, behavioral symptoms, and social adaptation difficulties among Tibetan college students in China’s high-altitude regions across different categories of soy product consumption (*χ*^2^ = 64.179, 45.118, 50.165; *p <* 0.001). Comparisons among different categories of physical exercise duration also revealed statistically significant differences in the prevalence rates of the three dimensions: emotional symptoms, behavioral symptoms, and social adaptation difficulties (*χ*^2^ = 114.849, 50.014, 48.109, *p <* 0.001). The comparative analysis results for different dimensions among male and female Tibetan college students in China’s high-altitude regions are presented in Table 3.

**Table 3:** Univariate analysis of soy product consumption, duration of physical exercise, and psychological symptoms among Tibetan college students in China’s high-altitude regions.

Sex/Category	Group	Number	Emotional symptoms			Behavioral symptoms			Social adaptation difficulties			Psychological symptoms		
			*N* (%)	*χ*^2^-value	*p*-value	*N* (%)	*χ*^2^-value	*p*-value	*N* (%)	*χ*^2^-value	*p*-value	*N* (%)	*χ*^2^-value	*p*-value
Boys														
Soy product consumption	≤2 times/day	1,130	234 (20.7)	33.477	<0.001	231 (20.4)	22.971	<0.001	222 (19.6)	44.321	<0.001	224 (19.8)	43.298	<0.001
	3–5 times/day	1,268	165 (13.0)			179 (14.1)			161 (12.7)			163 (12.9)		
	≥5 times/day	721	91 (12.6)			97 (13.5)			66 (9.2)			68 (9.4)		
Duration of physical exercise	<30 min/days	2062	395 (19.2)	54.655	<0.001	371 (18.0)	13.636	0.001	336 (16.3)	18.631	<0.001	367 (17.8)	51.525	<0.001
	30–60 min/day	761	70 (9.2)			100 (13.1)			86 (11.3)			69 (9.1)		
	>60 min/days	296	25 (8.4)			36 (12.2)			27 (9.1)			19 (6.4)		
Girls														
Soy product consumption	≤2 times/day	1,606	369 (23.0)	37.370	<0.001	355 (22.1)	27.078	<0.001	279 (17.4)	12.934	<0.001	346 (21.5)	40.585	<0.001
	3–5 times/day	1,575	312 (19.8)			312 (19.8)			213 (13.5)			291 (18.5)		
	≥5 times/day	770	95 (12.3)			101 (13.1)			98 (12.7)			83 (10.8)		
Duration of physical exercise	<30 min/days	3,181	695 (21.8)	50.969	<0.001	674 (21.2)	32.401	<0.001	521 (16.4)	30.446	<0.001	648 (20.4)	51.497	<0.001
	30–60 min/day	503	49 (9.7)			65 (12.9)			54 (10.7)			42 (8.3)		
	>60 min/days	267	32 (12.0)			29 (10.9)			15 (5.6)			30 (11.2)		
Total														
Soy product consumption	≤2 times/day	2,736	603 (22.0)	64.179	<0.001	586 (21.4)	45.118	<0.001	501 (18.3)	50.165	<0.001	570 (20.8)	81.274	<0.001
	3–5 times/day	2,843	477 (16.8)			491 (17.3)			374 (13.2)			454 (16.0)		
	≥5 times/day	1,491	186 (12.5)			198 (13.3)			164 (11.0)			151 (10.1)		
Duration of physical exercise	<30 min/days	5,243	1,090 (20.8)	114.849	<0.001	1,045 (19.9)	50.014	<0.001	857 (16.3)	48.109	<0.001	1,015 (19.4)	109.892	<0.001
	30–60 min/day	1,264	119 (9.4)			165 (13.1)			140 (11.1)			111 (8.8)		
	>60 min/days	563	57 (10.1)			65 (11.5)			42 (7.5)			49 (8.7)		

Table 4 presents the binary logistic regression analysis of soy product consumption, duration of physical exercise, and psychological symptoms among Tibetan college students in China’s high-altitude regions. After stratifying by sex, this study employed the presence (Yes = 1, No = 0) of psychological symptoms among Tibetan college students in high-altitude areas of China as the dependent variable. Binary logistic regression analyses were conducted using different levels of soy product consumption (≥5 times/days = 1, 3–5 times/day = 2, ≤2 times/day = 3) and duration of physical exercise (>60 min/days = 1, 30–60 min/day = 2, <30 min/day = 3) as independent variables. The overall analysis after adjusting for relevant covariates showed that compared with Tibetan college students consuming soy products ≥5 times/day, those consuming soy products 3–5 times/day (OR = 1.42, 95% CI: 1.16–1.73) and those consuming soy products ≤2 times/day (OR = 1.69, 95% CI: 1.39–2.07) had a higher risk of psychological symptoms compared to those consuming soy products ≥5 times/day (*p <* 0.001). Compared with Tibetan college students who exercised for >60 min/day, those exercising for <30 min/day (OR = 2.17, 95% CI: 1.59–2.96) also had a higher risk of psychological symptoms (*p <* 0.001). Analysis results stratified by sex for males and females are shown in Table 4.

**Table 4:** Binary logistic regression analysis of soy product consumption, duration of physical exercise, and psychological symptoms among Tibetan college students in China’s high-altitude regions.

Sex	Variable	Group	Model 1		Model 2		Model 3	
			OR (95% CI)	*p-*value	OR (95% CI)	*p-*value	OR (95% CI)	*p-*value
Boys	Soy product consumption	≥5 times/day	1.00		1.00		1.00	
		3–5 times/day	1.42 (1.05 ~ 1.91)	0.022	1.38 (1.02 ~ 1.86)	0.036	1.10 (0.81 ~ 1.51)	0.537
		≤2 times/day	2.37 (1.78 ~ 3.17)	<0.001	2.21 (1.65 ~ 2.95)	<0.001	1.48 (1.09 ~ 2.01)	0.012
	Duration of physical exercise	>60 min/days	1.00		1.00		1.00	
		30–60 min/day	1.45 (0.86 ~ 2.46)	0.164	1.49 (0.88 ~ 2.53)	0.139	1.53 (0.89 ~ 2.65)	0.126
		<30 min/days	3.16 (1.96 ~ 5.09)	<0.001	3.07 (1.9 ~ 4.96)	<0.001	3.08 (1.87 ~ 5.07)	<0.001
Girls	Soy product consumption	≥5 times/day	1.00		1.00		1.00	
		3–5 times/day	1.88 (1.45 ~ 2.44)	<0.001	1.80 (1.38 ~ 2.34)	<0.001	1.58 (1.21 ~ 2.06)	0.001
		≤2 times/day	2.27 (1.76 ~ 2.94)	<0.001	2.12 (1.63 ~ 2.75)	<0.001	1.64 (1.25 ~ 2.15)	<0.001
	Duration of physical exercise	>60 min/days	1.00		1.00		1.00	
		30–60 min/day	0.72 (0.44 ~ 1.18)	0.192	0.69 (0.42 ~ 1.14)	0.147	0.64 (0.38 ~ 1.06)	0.080
		<30 min/days	2.02 (1.37 ~ 2.98)	<0.001	1.84 (1.24 ~ 2.73)	0.002	1.51 (1.01 ~ 2.26)	0.047
Total	Soy product consumption	≥5 times/day	1.00		1.00		1.00	
		3–5 times/day	1.69 (1.39 ~ 2.05)	<0.001	1.63 (1.34 ~ 1.99)	<0.001	1.42 (1.16 ~ 1.73)	0.001
		≤2 times/day	2.34 (1.93 ~ 2.83)	<0.001	2.20 (1.81 ~ 2.67)	<0.001	1.69 (1.39 ~ 2.07)	<0.001
	Duration of physical exercise	>60 min/days	1.00		1.00		1.00	
		30–60 min/day	1.01 (0.71 ~ 1.44)	0.956	0.99 (0.70 ~ 1.41)	0.954	0.94 (0.66 ~ 1.35)	0.739
		<30 min/days	2.52 (1.86 ~ 3.4)	<0.001	2.40 (1.77 ~ 3.24)	<0.001	2.17 (1.59 ~ 2.96)	<0.001

OR, Odds Ratio; 95% CI, 95% Confidence Interval.

**Figure 2:**
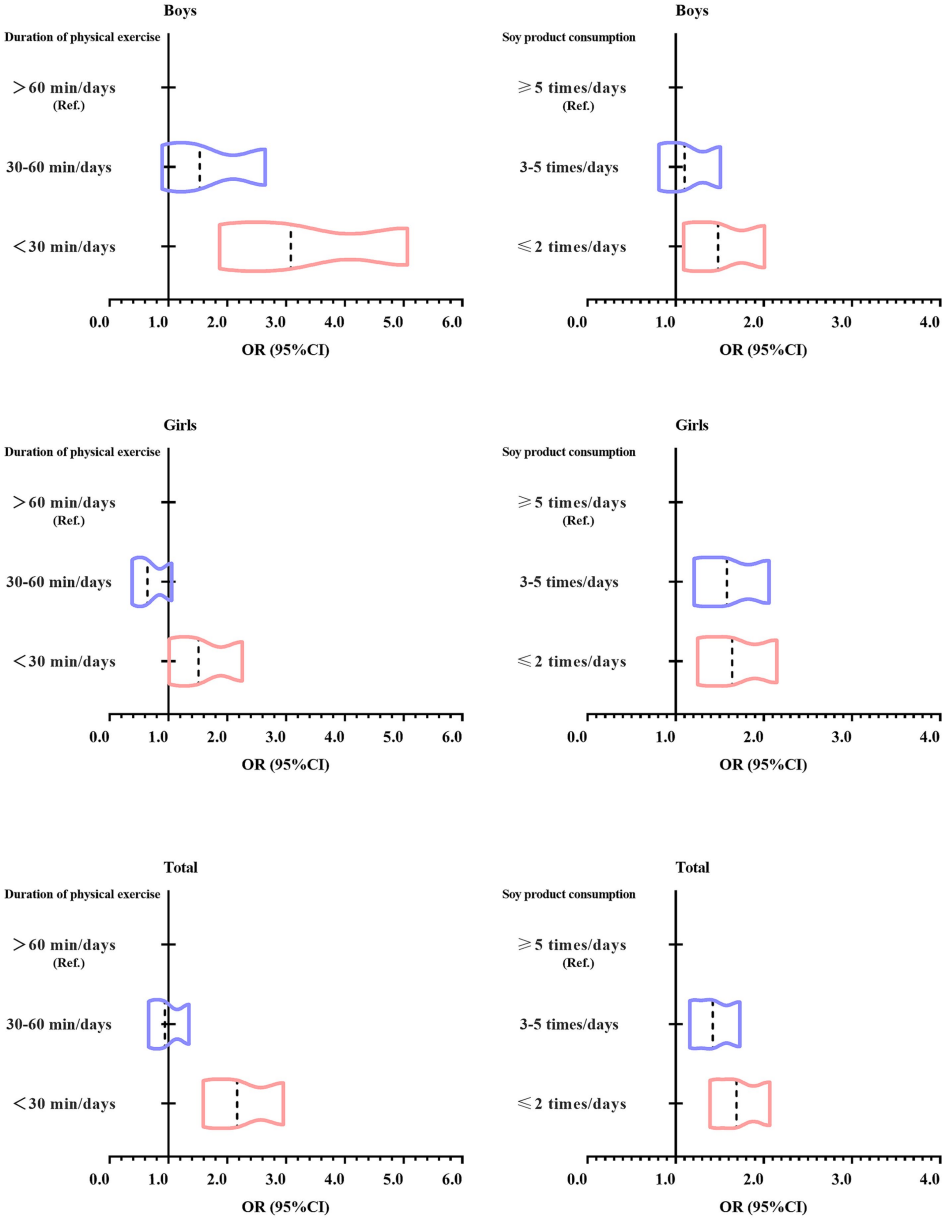
Trends in odds ratio changes from binary logistic regression analysis of soy product consumption, duration of physical exercise, and psychological symptoms among Tibetan college students in China’s high-altitude regions.

Figure 2 shows the trend in odds ratio (OR) values from a binary logistic regression analysis examining soy product consumption, duration of physical exercise, and psychological symptoms among Tibetan college students in China’s high-altitude regions. The figure indicates that as soy product consumption and duration of physical exercise decrease, the risk of developing psychological symptoms gradually increases, with the OR values shifting to the right.

Table 5 presents the binary logistic regression analysis of generalized linear models for soy product consumption, duration of physical exercise, and psychological symptoms among Tibetan college students in China’s high-altitude regions. After stratifying by sex, this study employed the presence (Yes = 1, No = 0) of psychological symptoms among Tibetan college students in high-altitude areas of China as the dependent variable. Bivariate logistic regression analyses using generalized linear models were conducted with different combinations of soy product consumption (≥5 times/day, 3–5 times/day, ≤2 times/day) and duration of physical exercise (>60 min/day, 30–60 min/day, <30 min/day) as independent variables. The overall analysis after covariate adjustment revealed that, compared with the reference group (soy product consumption ≥5 times/day and duration of physical exercise >60 min/day), the group with soy product consumption ≤2 times/day and duration of physical exercise <30 min/day had the highest risk of depressive symptoms (OR = 4.32, 95% CI: 2.49–7.51) (*p <* 0.001). Results of the stratified analysis by sex are presented in Table 5.

**Table 5:** Binary logistic regression analysis of soy product consumption, duration of physical exercise, and psychological symptoms in Tibetan college students in China’s high-altitude regions using a generalized linear model.

Sex	Classification of interaction		*OR* (95% *CI*)	*p*-value
	Soy product consumption	Duration of physical exercise		
Boys	≥5 times/day	>60 min/days	1.00	
		30–60 min/day	0.55 (0.19 ~ 1.56)	0.258
		<30 min/days	1.85 (0.82 ~ 4.21)	0.141
	3–5 times/day	>60 min/days	0.68 (0.21 ~ 2.21)	0.518
		30–60 min/day	1.96 (0.84 ~ 4.54)	0.119
		<30 min/days	2.06 (0.93 ~ 4.55)	0.074
	≤2 times/day	>60 min/days	1.01 (0.34 ~ 3.00)	0.984
		30–60 min/day	1.22 (0.51 ~ 2.92)	0.658
		<30 min/days	4.48 (2.04 ~ 9.83)	<0.001
Girls	≥5 times/day	>60 min/days	1.00	
		30–60 min/day	1.46 (0.53 ~ 4.00)	0.462
		<30 min/days	1.80 (0.80 ~ 4.03)	0.157
	3–5 times/day	>60 min/days	0.81 (0.23 ~ 2.88)	0.745
		30–60 min/day	1.42 (0.58 ~ 3.49)	0.449
		<30 min/days	3.51 (1.61 ~ 7.66)	0.002
	≤2 times/day	>60 min/days	3.44 (1.37 ~ 8.61)	0.008
		30–60 min/day	0.95 (0.37 ~ 2.46)	0.913
		<30 min/days	4.27 (1.96 ~ 9.29)	<0.001
Total	≥5 times/day	>60 min/days	1.00	
		30–60 min/day	0.85 (0.41 ~ 1.74)	0.648
		<30 min/days	1.82 (1.02 ~ 3.23)	0.042
	3–5 times/day	>60 min/days	0.73 (0.31 ~ 1.74)	0.482
		30–60 min/day	1.74 (0.94 ~ 3.20)	0.076
		<30 min/days	2.87 (1.65 ~ 5.00)	<0.001
	≤2 times/day	>60 min/days	2.09 (1.06 ~ 4.14)	0.034
		30–60 min/day	1.11 (0.59 ~ 2.11)	0.744
		<30 min/days	4.32 (2.49 ~ 7.51)	<0.001

OR, Odds Ratio; 95% CI, 95% Confidence Interval.

## 4 Discussion

With the continuous rise in lifestyle pressures, employment stress, and academic burdens, the prevalence of psychological symptoms among college students has shown a persistent upward trend, becoming one of the public health issues of common concern worldwide (Du et al., 2022). To our knowledge, this study represents the first analysis examining the association between soy product consumption, duration of physical exercise, and psychological symptoms among Tibetan college students in China’s high-altitude regions. The findings indicate that the prevalence of psychological symptoms among Tibetan college students in high-altitude areas of China is 16.6%. This rate is lower compared to survey results from Indian college students (34.8%) (Ts et al., 2017). The prevalence of psychological symptoms among Tibetan college students in China’s high-altitude regions is relatively low. Multiple factors contribute to this low prevalence. First, Tibetan college students in these high-altitude areas primarily reside in Tibet and Qinghai provinces in western China. In these regions, academic and employment pressures on college students are comparatively lower, which may be a key reason for the low prevalence of psychological symptoms observed in this study’s high-altitude Tibetan student population. Secondly, Tibetan college students who have lived long-term in high-altitude regions have developed unique physiological adaptation mechanisms to the oxygen-deprived environment. They exhibit enhanced cardiopulmonary function and blood oxygen saturation levels. These adaptive traits mitigate physiological stress responses caused by hypoxia, thereby reducing the prevalence of psychological symptoms among Tibetan college students (Li et al., 2024). Third, influenced by Buddhist culture, Tibetan college students emphasize compassion and patience. This cultural background fosters resilient and optimistic psychological qualities among them, thereby enhancing their psychological resilience and reducing the prevalence of psychological symptoms. However, the results are not entirely consistent. Compared to survey findings among American college students (15%), the results of this study are relatively higher (Hunt and Eisenberg, 2010). The variation in outcomes may stem from differences in the populations studied across various investigations. Additionally, the use of different questionnaires to assess psychological symptoms across studies could be a significant factor contributing to the divergence in research findings.

The findings of this study indicate that soy product consumption is associated with psychological symptoms among Tibetan college students in China’s high-altitude regions, with higher soy product consumption linked to a lower prevalence of psychological symptoms. Tao et al.’s research also demonstrated that soy product consumption is associated with a lower prevalence of psychological symptoms, consistent with the conclusions of this study (Zhang et al., 2022). The underlying causes are multifaceted. Primarily, the isoflavones in soybeans and their intestinal metabolites can effectively regulate the expression of brain-derived neurotrophic factor (BDNF) in the human hippocampus by crossing the blood–brain barrier. This enhances neural plasticity, thereby reducing the prevalence of psychological symptoms among Tibetan college students in high-altitude regions (Yu et al., 2018). Secondly, as phytoestrogens, isoflavones can mildly activate or antagonize central estrogen receptors, regulate serotonin (5-HT) and dopamine pathways, and enhance emotional stability among Tibetan college students in high-altitude regions. This enables them to better regulate their emotions when encountering psychological challenges, thereby preventing the onset of psychological symptoms (Miyake et al., 2018). On the other hand, higher soy product consumption can effectively lower blood pressure and improve lipid profiles, thereby reducing the risk of atherosclerosis. Cardiovascular health exhibits a significant negative correlation with the occurrence of psychological symptoms, thus indirectly lowering the prevalence of psychological symptoms among Tibetan college students in high-altitude regions (Smiley et al., 2017).

The findings of this study also indicate that a correlation exists between the duration of physical exercise and psychological symptoms among Tibetan college students in China’s high-altitude regions. Compared to those with shorter durations of physical exercise, Tibetan college students who engaged in longer durations of physical exercise exhibited lower prevalence rates of psychological symptoms. This suggests that the association between the duration of physical exercise and psychological symptoms among college students remains consistent regardless of altitude. In high-altitude regions, engaging in active physical exercise duration also plays a positive role in reducing psychological symptoms among Tibetan college students. Deng’s research indicates that maintaining 60 min of moderate-to-vigorous physical activity (MVPA) daily has a positive effect on lowering the prevalence of psychological symptoms (Deng et al., 2024). Zhang et al.’s research also confirmed this conclusion (Zhang et al., 2025). The reasons lie in several aspects. First, regular physical exercise effectively enhances college students’ cardiopulmonary function, offering the same benefits to Tibetan students in high-altitude regions. Research indicates that active exercise among college students in high-altitude areas can significantly improve brain activation levels, thereby effectively promoting executive function and providing protective effects against the occurrence of psychological symptoms (Sun et al., 2025). Secondly, regular physical exercise can improve sleep quality among college students in high-altitude regions, reducing the incidence of sleep disorders and difficulty falling asleep. Better sleep quality plays a positive role in alleviating students’ emotional distress and psychological symptoms (Gupta et al., 2022). Research has also found that regular, vigorous physical exercise can help the body effectively regulate the hypothalamic–pituitary–adrenal (HPA) axis response, reduce cortisol levels at rest, lessen physiological stress reactions, and positively contribute to emotional stability in exercisers (Heijnen et al., 2015).

This study further analyzed the association between the combined effects of soy product consumption and duration of physical exercise and psychological symptoms among Tibetan college students in China’s high-altitude regions. The results indicate that higher frequency of soy product consumption and longer duration of physical exercise are associated with a reduced prevalence of psychological symptoms among Tibetan college students in high-altitude areas. Furthermore, this association remains consistent regardless of sex.

This study possesses certain strengths and limitations. Regarding strengths: First, to our knowledge, this study is the first to analyze the association between soy product consumption, duration of physical exercise, and psychological symptoms among Tibetan college students in China’s high-altitude regions. The findings may provide insights for the prevention and intervention of psychological symptoms among Tibetan college students in China’s high-altitude areas, and potentially for college students in high-altitude regions worldwide. Second, this study analyzed the relationship between soy product consumption, duration of physical exercise, and psychological symptoms among Tibetan college students in China’s high-altitude regions using a relatively large participant sample, ensuring a degree of representativeness. However, this study also has certain limitations. First, this study is a cross-sectional survey, capable only of analyzing the association between soy product consumption, duration of physical exercise, and psychological symptoms, rather than establishing causal relationships. Prospective cohort studies should be conducted in the future to address this limitation. Second, the limited number of covariates included in this study may have influenced the results. Future studies should incorporate additional factors influencing psychological symptoms, such as smoking, alcohol consumption, dietary behaviors, and family environment, to better analyze the existing associations. Third, this study assessed soy product consumption, duration of physical exercise, and psychological symptoms using participant self-report questionnaires, which inevitably introduces some bias compared to actual conditions. Future research could employ more objective assessment methods to enhance the accuracy of results.

## 5 Conclusion

There is an association between soy product consumption, duration of physical exercise, and psychological symptoms among Tibetan college students in China’s high-altitude regions. Individuals with lower soy product consumption and shorter duration of physical exercise exhibit a lower prevalence of psychological symptoms. Furthermore, this association remains consistent regardless of sex. Soy product consumption and duration of physical exercise may be significant factors influencing psychological symptoms among Tibetan college students in high-altitude regions. This study provides essential theoretical support for the prevention and intervention of psychological symptoms among Tibetan college students in China’s high-altitude areas and college students in high-altitude regions worldwide.

## Data Availability

The data analyzed in this study is subject to the following licenses/restrictions: to protect the privacy of participants, the questionnaire data will not be disclosed to the public. If necessary, you can contact the corresponding author. Requests to access these datasets should be directed to ojuistgh@163.com.
